# Discovery and characterization of a sulfoquinovose mutarotase using kinetic analysis at equilibrium by exchange spectroscopy

**DOI:** 10.1042/BCJ20170947

**Published:** 2018-04-16

**Authors:** Palika Abayakoon, James P. Lingford, Yi Jin, Christopher Bengt, Gideon J. Davies, Shenggen Yao, Ethan D. Goddard-Borger, Spencer J. Williams

**Affiliations:** 1 School of Chemistry, University of Melbourne, Parkville, VIC 3010, Australia; 2 Bio21 Molecular Science and Biotechnology Institute, University of Melbourne, Parkville, VIC 3010, Australia; 3 ACRF Chemical Biology Division, The Walter and Eliza Hall Institute of Medical Research, Parkville, VIC 3052, Australia; 4 Department of Medical Biology, University of Melbourne, Parkville, VIC 3010, Australia; 5 York Structural Biology Laboratory, Department of Chemistry, University of York, Heslington YO10 5DD, U.K.

**Keywords:** enzymology, metabolism, mutarotase, NMR spectroscopy, sulfoglycolysis

## Abstract

Bacterial sulfoglycolytic pathways catabolize sulfoquinovose (SQ), or glycosides thereof, to generate a three-carbon metabolite for primary cellular metabolism and a three-carbon sulfonate that is expelled from the cell. Sulfoglycolytic operons encoding an Embden–Meyerhof–Parnas-like or Entner–Doudoroff (ED)-like pathway harbor an uncharacterized gene (*yihR* in *Escherichia coli*; *PpSQ1_00415* in *Pseudomonas putida*) that is up-regulated in the presence of SQ, has been annotated as an aldose-1-epimerase and which may encode an SQ mutarotase. Our sequence analyses and structural modeling confirmed that these proteins possess mutarotase-like active sites with conserved catalytic residues. We overexpressed the homolog from the sulfo-ED operon of *Herbaspirillum seropedicaea* (*Hs*SQM) and used it to demonstrate SQ mutarotase activity for the first time. This was accomplished using nuclear magnetic resonance exchange spectroscopy, a method that allows the chemical exchange of magnetization between the two SQ anomers at equilibrium. *Hs*SQM also catalyzed the mutarotation of various aldohexoses with an equatorial 2-hydroxy group, including d-galactose, d-glucose, d-glucose-6-phosphate (Glc-6-P), and d-glucuronic acid, but not d-mannose. *Hs*SQM displayed only 5-fold selectivity in terms of efficiency (*k*_cat_/*K*_M_) for SQ versus the glycolysis intermediate Glc-6-P; however, its proficiency [*k*_uncat_/(*k*_cat_/*K*_M_)] for SQ was 17 000-fold better than for Glc-6-P, revealing that *Hs*SQM preferentially stabilizes the SQ transition state.

## Introduction

**Figure 1.**
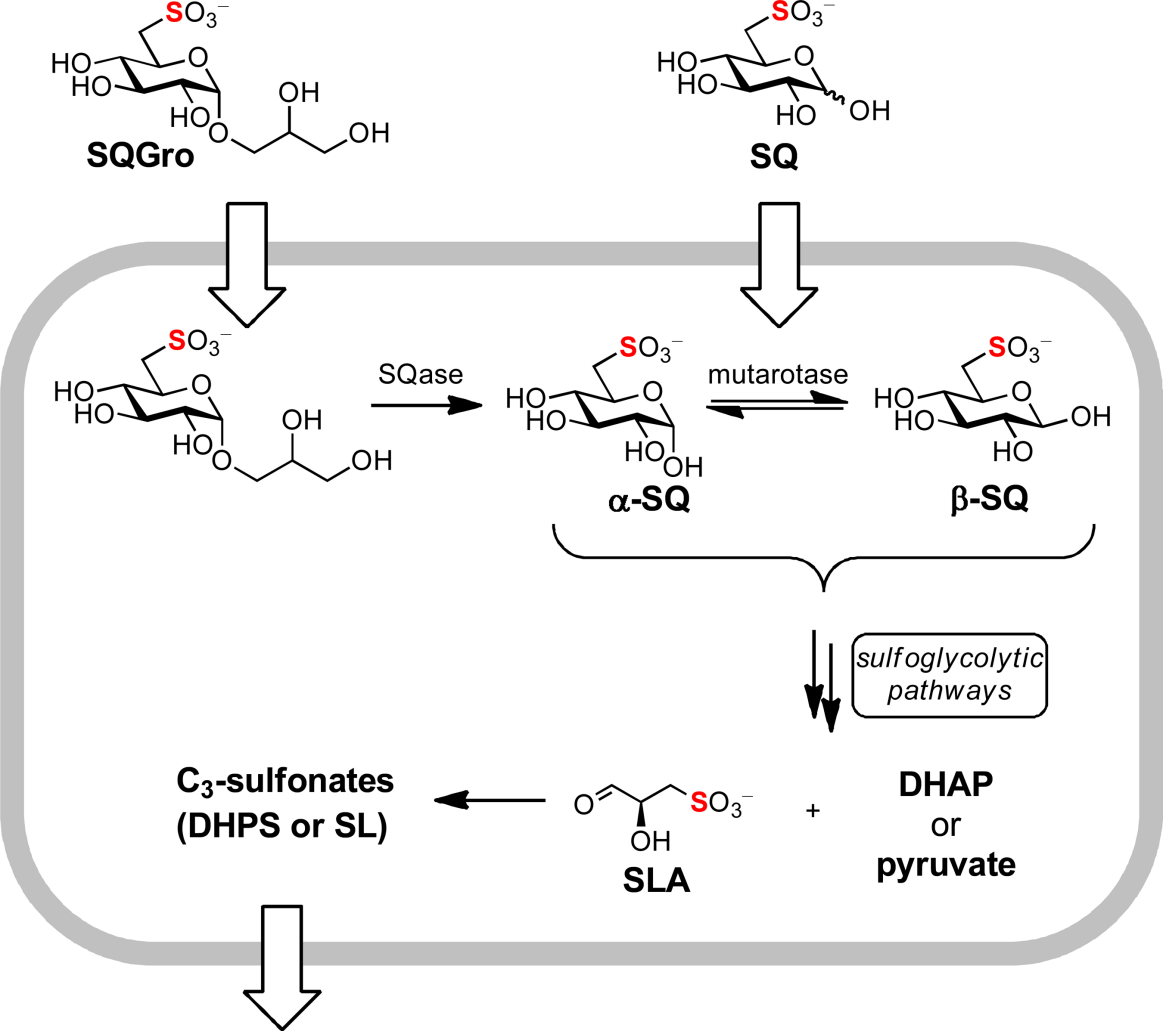
Summary of sulfoglycolysis. Importation of SQGro and cleavage by SQase, or direct importation of SQ, provides an intracellular pool of SQ anomers that can be interconverted by SQ mutarotase. SQ is metabolized by sulfo-ED or sulfo-EMP pathways to SLA and then to the C_3_-sulfonates DHPS or SL, prior to export.

Various prokaryotes metabolize the sugar sulfoquinovose (SQ) to sulfolactaldehyde (SLA) and dihydroxyacetone phosphate (DHAP), via an Embden–Meyerhof–Parnas (EMP)-like pathway [1], or pyruvate, via an Entner–Doudoroff (ED)-like pathway [2,3] (Figure 1). While the DHAP or pyruvate feed into primary metabolic pathways, SLA is converted into 2,3-dihydroxypropanesulfonate (DHPS) or sulfolactate (SL) by the sulfo-EMP and sulfo-ED pathways, respectively, and excreted from the cell. Yet, SQ is rarely encountered as a free sugar in nature; rather, it is liberated from the plant sulfolipid α-sulfoquinovosyl diacylglycerol (SQDG), or its delipidated form α-sulfoquinovosyl glycerol (SQGro), by the action of glycoside hydrolases termed sulfoquinovosidases (SQases) [4]. SQases are retaining glycosidases, and result in the initial formation of α-SQ, which can undergo mutarotation to β-SQ at an unknown rate. The anomeric preferences of the immediate downstream enzymes that utilize SQ (SQ isomerase for the sulfo-EMP pathway; SQ dehydrogenase for the sulfo-ED pathway) are unknown.

All proteins comprising a sulfo-ED or sulfo-EMP pathway are typically encoded within a single gene cluster. These clusters usually include an SQase, which highlights that SQ glycosides are important natural feedstocks for sulfoglycolytic pathways [1,2]. The gene clusters also encode a conserved uncharacterized protein, annotated as an aldose-1-epimerase, which likely catalyzes SQ mutarotation: an enzyme activity yet to be reported. Mutarotases are widely distributed enzymes that facilitate the rapid mutarotation of aldoses to enhance flux through metabolic pathways when enzymes acting on reducing sugars are specific for a single anomer [5,6].

A classical approach to studying mutarotases involves measuring rates of mutarotation by polarimetry [7–10]. These assays are limited by an inability to prepare pure samples of a single anomer of many reducing sugars. Indeed, to date, it has not been possible to obtain SQ as a single anomer, which makes this approach of limited use for studying putative SQ mutarotases (SQMs). Alternative approaches to studying mutarotases employ coupled assays in which only one anomer acts as a substrate for a secondary enzyme [11,12] or a chemical reaction (e.g. bromine oxidation [13]), or use a glycosidase to prepare a single anomer in solution prior to the addition of the mutarotase [12]. The high rate of uncatalyzed mutarotation under standard conditions often renders these assays technically demanding, and a requirement for different coupling enzymes for each substrate undermines their generality. An alternative approach is to study reaction rates at equilibrium. NMR (nuclear magnetic resonance) spectroscopy is ideally suited to this approach using the technique of exchange spectroscopy (EXSY) [14,15]. EXSY involves chemical shift labeling of the spin population of nuclei at one site within a substrate, followed by a chemical reaction that changes the chemical environment of individual nuclei resulting in magnetization transfer to the new site, and finally sampling of the magnetization states of the nuclei in the substrate and product. Because of the spectral resolution of NMR spectroscopy and the potential to conduct two-dimensional variants, EXSY can be used to study unidirectional reactions at equilibrium. Several reports have described the development of saturation transfer and inversion transfer NMR methods for analysis of equilibrium exchange rates for mutarotases [16–19].

Here, we disclose the first measurement of SQ mutarotase activity, using an enzyme from *Herbaspirillum seropedicaea* (*Hs*SQM) and 2D (two-dimensional) EXSY, and analyze its selectivity for various reducing sugars with and without an anionic substituent at C6. *Hs*SQM exhibited a broad spectrum of activity for sugars with an equatorial hydroxyl group at C2 and with, or without, charge at C6. 1D (one-dimensional) EXSY was used to measure reaction rates to obtain Michaelis–Menten kinetics for *Hs*SQM with SQ and d-glucose-6-phosphate(Glc-6-P), a common cytoplasmic metabolite, revealing an approximate 5-fold preference for SQ as a substrate. Sequence and structural analyses revealed that *Hs*SQM belongs to the galactose mutarotase-like Structural Classification of Proteins (SCOP) with strictly conserved active-site residues that are proposed to be involved in substrate binding and catalysis.

## Materials and methods

### Reagents

d-Glucose (Glc), d-mannose (Man), d-galactose (Gal), d-glucuronic acid (GlcA), and Glc-6-P were purchased from Sigma–Aldrich. d-Sulfoquinovose was purchased from MCAT GmbH (Germany, http://www.MCAT.de). 4-Nitrophenyl α-d-sulfoquinovoside and *Escherichia coli* YihQ sulfoquinovosidase have been described recently [4].

### Cloning, expression, and purification of HsSQM

A gene encoding the *H. seropedicaea* strain AU14040 protein WP_069374721.1 was codon-harmonized for *E. coli*, synthesized, and cloned into the pET29 vector using the *NdeI* and *XhoI* restriction sites to provide pET29-HsSQM (see Supplementary Information). This plasmid was transformed into chemically competent ‘NEB Express’ *E. coli*, plated onto LB agar (50 μg/ml kanamycin), and incubated at 37°C for 16 h. A single colony was used to inoculate 10 ml of LB media containing 50 μg/ml kanamycin followed by incubation at 37°C for 16 h. This culture was used to inoculate 1000 ml of ‘S-broth’ (35 g tryptone, 20 g yeast extract, and 5 g NaCl, pH 7.4) containing 50 μg/ml kanamycin, which was incubated with shaking (250 rpm) at 37°C until it reached an OD_600_ of 0.7. The culture was cooled to room temperature, IPTG was added to a final concentration of 100 μM, and then incubated with shaking (200 rpm) at 18°C for 19 h. The cells were harvested by centrifugation at 17 000 ***g*** for 20 min at 4°C and then resuspended in 40 ml of binding buffer (50 mM NaP_i_, 500 mM NaCl, and 5 mM imidazole, pH 7.5) containing a protease inhibitor (Roche complete EDTA-free protease inhibitor cocktail) and lysozyme (0.1 mg/ml) by nutating at 4°C for 30 min. Benzonase (1 μl) was added to the mixture and lysis was affected by sonication. The lysate was clarified by centrifugation (17 000 ***g*** for 20 min at 4°C), and the supernatant was filtered (0.45 μm) and loaded onto a 1 ml HiTrap TALON column (GE Healthcare). The column was washed with 15 ml of binding buffer and the protein was eluted using elution buffer (50 mM NaP_i_, 500 mM NaCl, and 500 mM imidazole, pH 7.5). Fractions containing the protein of interest (as determined by SDS–PAGE) were further purified by size exclusion chromatography on a HiLoad 16/600 Superdex 75 column using 50 mM NaP_i_ and 150 mM NaCl (pH 7.5). Fractions containing the protein of interest were further purified on a MonoQ 5/50 column with protein bound in buffer A (20 mM Tris, pH 7.5) and eluted with a linear gradient from 100% buffer A to 100% buffer B (20 mM Tris, NaCl 500 mM, pH 7.5) over 15 ml at 1 ml/min. Protein yield was ∼5 mg/l of culture.

### NMR magnetization-transfer experiments

The theoretical background of NMR for the study of the kinetics of chemical exchange has been well documented [14,20] and only a brief overview is presented. The interconversion of anomers by mutarotation is described by a simple two-state equilibrium:

1 $$\alpha \overset{k_{1}}{\underset{k_{-1}}{\;⇌}}\beta .$$

For a species exchanging between isomers, the dependence upon time (*t*) of the longitudinal nuclear magnetizations of corresponding nuclei from the α and β anomer spin populations (*I*_α_) and (*I*_β_), corresponding to the integrals, are given by the Bloch-McConnell equation:

2 $$\frac{d}{dt}\left(\begin{array}{c} I_{\alpha }\\ I_{\beta } \end{array}\right)=\left(\begin{array}{cc} -R_{\alpha }-k_{1} & k_{-1}\\ k_{1} & -R_{\beta }-k_{-1} \end{array}\right)\left(\begin{array}{c} I_{\alpha }\\ I_{\beta } \end{array}\right)+\left(\begin{array}{cc} R_{\alpha } & 0\\ 0 & R_{\beta } \end{array}\right)\left(\begin{array}{c} I_{\alpha }^{0}(t)\\ I_{\beta }^{0}(t) \end{array}\right),$$

where *R*_α_ and *R*_β_ are the longitudinal relaxation rates, characterizing the return of the magnetizations toward their respective equilibrium values, $I_{\alpha }^{0}$ and $I_{\beta }^{0}$; *k*_1_ and *k*_−1_ are the extrinsic rate constants for the forward and reverse reactions for each anomer, α and β, respectively. The solution of this equation gives the intensity of the transferred magnetization at *τ*_mix_:

3 $$\left(\begin{array}{c} I_{\alpha }(\tau_{\mathrm{mix}})\\ I_{\beta }(\tau_{\mathrm{mix}}) \end{array}\right)=\exp\left[\left(\begin{array}{cc} -R_{\alpha }-k_{1} & k_{-1}\\ k_{1} & -R_{\beta }-k_{-1} \end{array}\right)\tau_{\mathrm{mix}}\right]\times \left(\begin{array}{c} I_{\alpha }(0)-I_{\alpha }^{0}\\ I_{\beta }(0)-I_{\beta }^{0} \end{array}\right)+\left(\begin{array}{c} I_{\alpha }^{0}\\ I_{\beta }^{0} \end{array}\right).$$

Under the condition of chemical equilibrium, the net flux is zero. However, the unidirectional fluxes remain and are equal in both directions. Thus,

4 $$k_{1}{\left[\alpha \right]}^{\mathrm{eq}}\;=\;k_{-1}{\left[\beta \right]}^{\mathrm{eq}}$$

and

5 $$K_{\mathrm{eq}}\;=\;\frac{{\left[\beta \right]}^{\mathrm{eq}}}{{\left[\alpha \right]}^{\mathrm{eq}}}\;=\frac{k_{1}}{k_{-1}}=\exp\left(-\frac{\Delta G}{\mathrm{RT}}\right)\;.$$

To obtain the rate constants, *k*_1_ and *k*_−1_, at a given concentration ratio of the substrate and enzyme, a series of 1D ^1^H EXSY spectra with mixing time *τ*_mix_, ranging from 5 ms to 1.5 s, were acquired using a 1D selective NOESY pulse sequence. For each substrate concentration, the normalized integrals of the substrate (H1α) and product (H1β) anomeric signals are plotted against mixing time, providing buildup and decay curves, respectively, as described previously [21]. The data were then fitted to a second-order polynomial, an approximation to eqn (3), which is valid for short mixing times. The tangent at *τ*_mix_ = 0, the so-called initial rate approach [22], provides estimates of both exchange-rate constants *k*_1_ and *k*_−1_.

### Michaelis–Menten kinetics

The equilibrium exchange kinetics (reaction rate, *v*^eq^) of a mutarotase-mediated reaction can be expressed as follows:

6 $$v^{\mathrm{eq}}([\alpha ])=\;\frac{V_{\max}^{\mathrm{eq}}[\alpha ]}{K_{M}^{\mathrm{eq}}\;+[\alpha ]}.$$

$V_{\max}^{\mathrm{eq}}$ is the maximum rate of reaction, reached under saturating equilibrium exchange conditions when $[\alpha ]\gg K_{M}^{\mathrm{eq}}$. The rate of the mutarotation process can be expressed in terms of the Michaelis–Menten equation:

7 $$v^{\mathrm{eq}}=\;k_{1}[\alpha {]}^{\mathrm{eq}}=\;\frac{V_{\max}^{\mathrm{eq}}[\alpha ]}{K_{M}^{\mathrm{eq}}+[\alpha ]}.$$

*k*_cat_ is calculated by dividing $V_{\max}^{\mathrm{eq}}$ by the enzyme concentration.

### 2D ^1^H-^1^H EXSY experiments

2D ^1^H-^1^H EXSY spectra for all substrates (SQ, Glc-6-P, Glc, Gal, Man, GlcA) were acquired at 25°C on a Bruker Avance II 800 spectrometer equipped with a TXI cryoprobe using the standard 2D NOESY/EXSY pulse sequence (noesyphpr). Spectra were collected with four and eight scans in the absence and presence of HsSQM, respectively. A mixing time, *τ*_mix_ of 1.1 s, was used for all the experiments. Spectra were processed and analyzed using TOPSPIN (version 3.2 Bruker), and ^1^H chemical shifts were referenced indirectly to DSS (4,4-dimethyl-4-silapentane-1-sulfonic acid) at 0 ppm via the HOD resonance, 4.77 ppm at 25°C. Samples were prepared in D_2_O buffer consisting of 50 mM sodium phosphate, 150 mM NaCl (pD 7.5) with a substrate concentration 5 mM in the absence or presence of 1.51 µM *Hs*SQM. pD was measured using a pH meter and the relationship pD = pH + 0.4.

### 1D ^1^H EXSY experiments

#### Michaelis–Menten parameters

1D ^1^H EXSY spectroscopic studies for SQ and Glc-6-P were performed at 25°C on a Bruker Avance III 600 spectrometer equipped with a TCI cryoprobe using a 1D selective NOESY pulse sequence (selnogp, Bruker). Samples were prepared in D_2_O buffer consisting of 50 mM sodium phosphate, 150 mM NaCl (pD 7.5) using 1.50 µM *Hs*SQM at substrate concentrations ranging from 0.5 to 15.0 mM (for SQ) or 0.5–30.0 mM (for Glc-6-P). pD values were calculated using the following relationship: pD = pH + 0.4. For each sample, 1D ^1^H EXSY spectra with mixing time, *τ*_mix_, ranging from 5 ms to 1.5 s were acquired with the number of scans varying between 32 and 256 depending on the concentration of substrate. A recycle delay of 13.4 s (∼3–5 times the measured ^1^H *T*_1_ relaxation times) between scans was used for the acquisition of 1D ^1^H EXSY spectra. Spectra were subsequently processed and analyzed using TOPSPIN (version 3.2 Bruker). Kinetic data for the conversion of the β-anomer of SQ to the α-anomer were obtained by selectively inverting the resonance of H5β at 3.72 ppm using a Gaussian-shaped pulse of 20 ms, and *vice versa*, for conversion of the α-anomer to the β-anomer, the same selective pulse was applied to the resonance of H5α at 4.15 ppm. Similarly, for the conversion of the β-anomer of Glc-6-P to the α-anomer, kinetic data were obtained by selectively inverting the signal for H1β at 4.57 ppm. A buildup curve for the β-anomer could not be obtained since the signal for H1β at 4.57 ppm was affected by the nearby HOD peak at 4.77 ppm (see Supplementary Data for representative ^1^H NMR spectra). Instead, the rate constant for the conversion of the α-anomer to the β-anomer was calculated using eqn (4). Rates for each concentration were calculated using the Prism 6 software package (GraphPad Scientific Software). Data were fitted to a second-order polynomial function as described previously by Aski et al. [21].

#### pD dependence of activity for HsSQM

The Michaelis–Menten parameter *k*_cat_/*K*_M_ was measured for Glc-6-P mutarotation in D_2_O buffer consisting of 50 mM citrate/phosphate and 150 mM NaCl at a range of pD values (5.6, 6.1, 6.5, 7.0, 7.5, 8.1, 9.0, 9.4, 9.8, 10.4, and 10.9) at 25°C. Reactions were initiated by adding 1.48–2.96 µM *Hs*SQM to Glc-6-P (5.0 mM) in buffer and the rate was measured by 1D ^1^H EXSY as described above with mixing time, *τ*_mix_, ranging from 5 ms to 1.0 s and the number of scans from 64 and 128. Kinetic data were obtained for the conversion of the β-anomer of Glc-6-P to the α-anomer, by selectively inverting the signal for H1β at 4.57 ppm. *k*_cat_/*K*_M_ and p*K*_a_ values were calculated using the Prism 6 software package (Graphpad Scientific Software). pH dependence was fit to the following function:

8 $$y=\;\frac{k_{\mathrm{cat}}}{K_{M}}\left[\left(\frac{1}{1+((10^{-\mathrm{pH}}/10^{-pKa1})+(10^{-pKa2}/10^{-\mathrm{pH}}))}\right)\right]+c.$$

### Uncatalyzed mutarotation rate measurement

The uncatalyzed rate constant for SQ mutarotation was measured using polarimetry on a Jasco DIP-1000 digital polarimeter equipped with Na 589 nm lamp and 100.00 mm cell, using a sulfoquinovosidase to generate α-SQ from 4-nitrophenyl α-d-sulfoquinovoside (PNPSQ). Analysis by ^1^H NMR spectroscopy and thin layer chromatography revealed that hydrolysis of PNPSQ was complete within 5 min. SQase (final concentration 2.13 µM) was added to a solution of PNPSQ (final concentration 12.4 mM) in buffer in a final volume of 2 ml. Buffers consisted of 50 mM sodium phosphate, 150 mM NaCl in H_2_O (pH 7.1); 50 mM sodium phosphate, 150 mM NaCl in D_2_O (pD 7.5); or 10–50 mM sodium phosphate, 2 M NaCl in D_2_O (pD 7.5). The reaction mixture was transferred to the polarimetry cell, and, after 5 min, the mutarotation rate was monitored continuously at 26 ± 1°C by reading specific rotation at various times. The rate constant was calculated using the Prism 6 software package (Graphpad Scientific Software). Data were fitted to a one-phase decay function, *t*_1/2_ = ln(2)/*k*.

For mutarotation, as described in eqn (1), the rate of change in the concentration of the α-anomer has two contributions: it is depleted by the forward reaction at rate *k*_1_[α] and is replenished by the reverse reaction at rate *k*_−1_[α]. The net rate of change is therefore:

9 $$\frac{d[\alpha ]}{dt}\;=\;-k_{1}[\alpha ]+k_{-1}[\beta ].$$

The solution of this first-order differential equation is:

10 $$[\alpha ]\;=\;\left\{\frac{k_{-1}+k_{1}e^{-(k_{1}+k_{-1})t}}{k_{1}+k_{-1}}\right\}[\alpha {]}_{0}$$

and thus the first-order decay constant (*k*) is related to the forward and reverse rates as follows:

11 $$k\;=\;k_{1}+k_{-1}.$$

Using eqn (5), which relates the equilibrium constant to the forward and reverse rates, this shows that:

12 $$K^{\mathrm{eq}}=\;\frac{{\left[\beta \right]}^{\mathrm{eq}}}{{\left[\alpha \right]}^{\mathrm{eq}}}\;=\frac{k_{1}}{k-k_{1}}.$$

## Results

**Figure 2.**
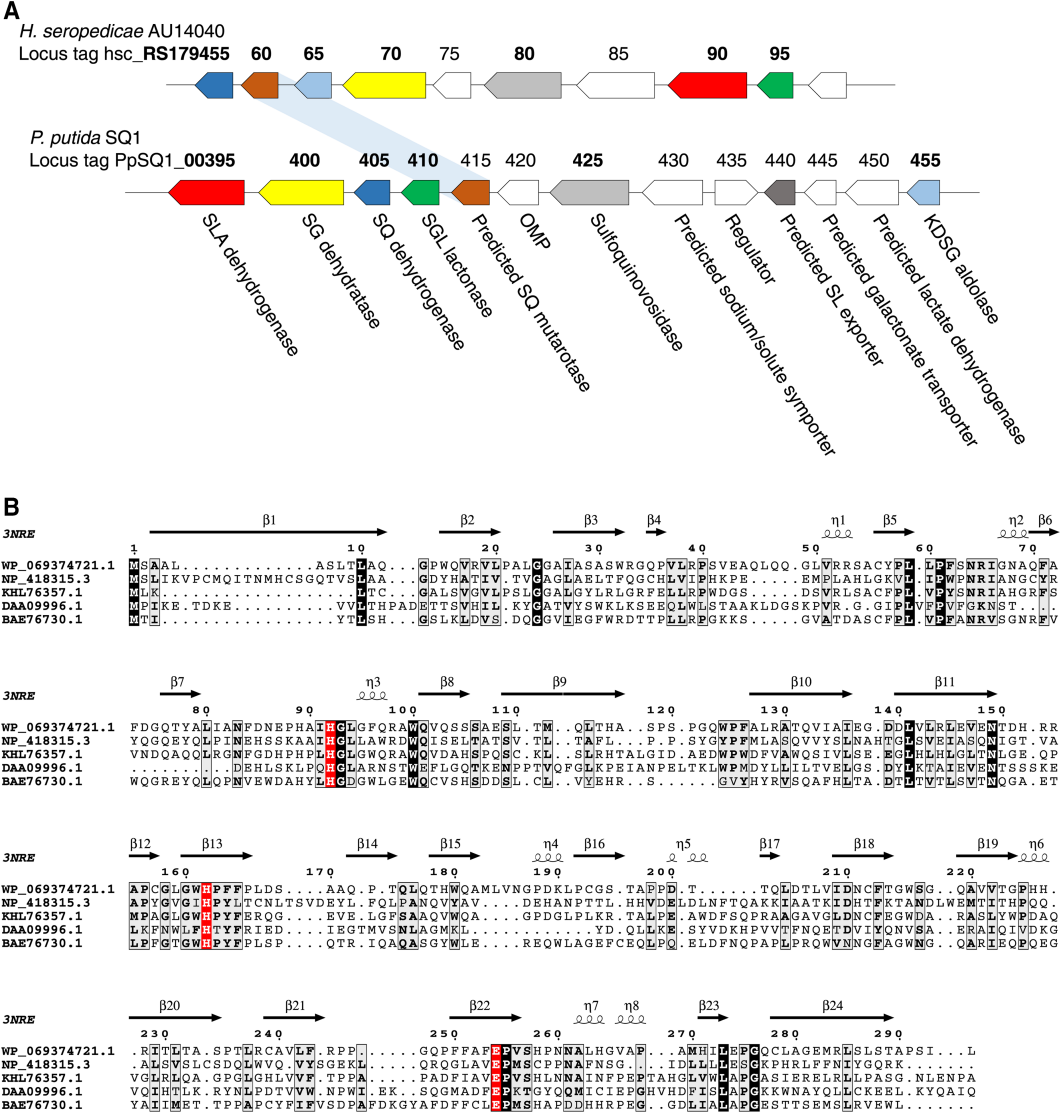
*H. seropedicaea* contains a sulfo-ED operon and a putative SQ mutarotase. (**A**) Operon structure of *P. putida* SQ1 and *H. seropedicaea* strain AU14040. Bold indicates genes for which enzymatic activity has been biochemically determined in at least one organism. (**B**) Alignment of various putative mutarotases with secondary structural elements. WP_069374721.1, *Hs*QM from *H. seropedicaea*; NP_418315.3, YihQ from *E. coli*; KHL76357.1, PpSQ1_00415 from *Pseudomonas putida* SQ1; DAA09996.1, YMR099C hexose-1-phosphate mutarotase from *S. cerevisiae*; BAE76730.1, YphB aldose-1-epimerase from *E. coli* (BAE76730.1). The secondary structural elements are annotated from the structure of *E. coli* YphB (PDB 3nre).

Our initial efforts to characterize SQ mutarotases focused on expressing *YihR* from *E. coli* (which utilizes the sulfo-EMP pathway) [1] and *PpSQ1_00415* from *Pseudomonas putida* SQ1 (which utilizes the sulfo-ED pathway) [2] in an *E. coli* expression system. However, despite screening several expression constructs and conditions, only a poor yield of low-quality protein was ever obtained. Thus, we turned our attention to other putative SQ mutarotases from various bacteria that possess sulfoglycolytic gene clusters and succeeded in obtaining WP_069374721.1 (hereafter *Hs*SQM) from *H. seropedicaea* in high yield and purity. *H. seropedicaea* is a nitrogen-fixing endophytic bacterium capable of colonizing the intercellular spaces of grasses such as rice and sugar cane [23] and contains a predicted sulfo-ED operon analogous to that of *P. putida* SQ1 but lacking in synteny (Figure 2A) [2]. A sequence alignment of these three putative sulfoquinovose mutarotases, as well as two structurally characterized hexose mutarotases, is provided in Figure 2B: *Hs*SQM shares 37% similarity (19% identity) with *E. coli* YihR and 50% similarity (35% identity) with *P. putida* SQ1 PpSQ1_00415 (Supplementary Table S1). All three proteins retain the highly conserved residues of other hexose mutarotases (His92, His162, and Glu254 in *Hs*SQM). The equivalent residue to *Hs*SQM His162 in galactose mutarotase from *E. coli* has previously been proposed to be involved in substrate binding [24], whereas the equivalent residues to His92 and Glu254 are proposed to act in roles of general acid and general base, respectively, in the first half of the reaction leading to the acyclic aldehyde, in galactose mutarotases from both *Lactococcus lactis* and *E. coli* [24,25].

**Figure 3.**
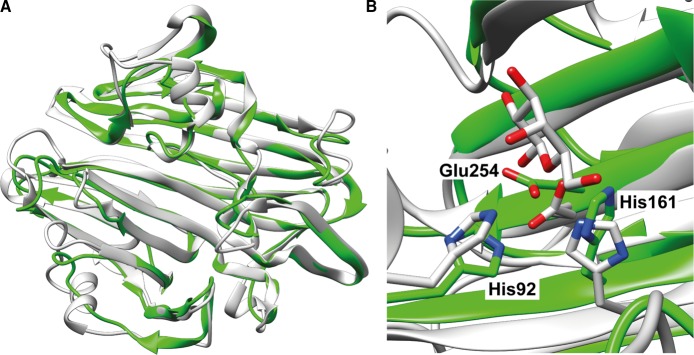
Homology model of *Hs*SQM. (**A**) Overlay of *Hs*SQM homology model (green) with a putative mutarotase from *C. acetobutylicum* (PDB 3OS7; gray). (**B**) The active sites of the *Hs*SQM homology model (green) overlaid with the galactose mutarotase domain of gal10 from *S. cerevisae* with d-galactose bound (gray, PDB 1Z45). Residue numbers are for *Hs*SQM.

The iTASSER server [26–28] provided a homology model of *Hs*SQM with a *C*-score of 0.87, suggesting it to be a good approximation for the protein's native fold. This was compared with existing structures by using TM-align [29,30] and returned only other (predicted) mutarotase structures. The greatest structural similarity was found between the *Hs*SQM model and a galactose mutarotase-like protein from *Clostridium acetobutylicum* (PDB 3OS7, Figure 3A), with an RMSD of 1.55 Å between structural models, despite both proteins sharing <15% identity. Structural alignment of the *Hs*SQM homology model and the galactose mutarotase domain of gal10 from *S. cerevisae* with d-galactose bound (PDB 1Z45) [31] reveals that the conserved active-site residues His92, His162, and Glu254 in the homology model are positioned appropriately for catalysis (Figure 3B).

**Figure 4.**
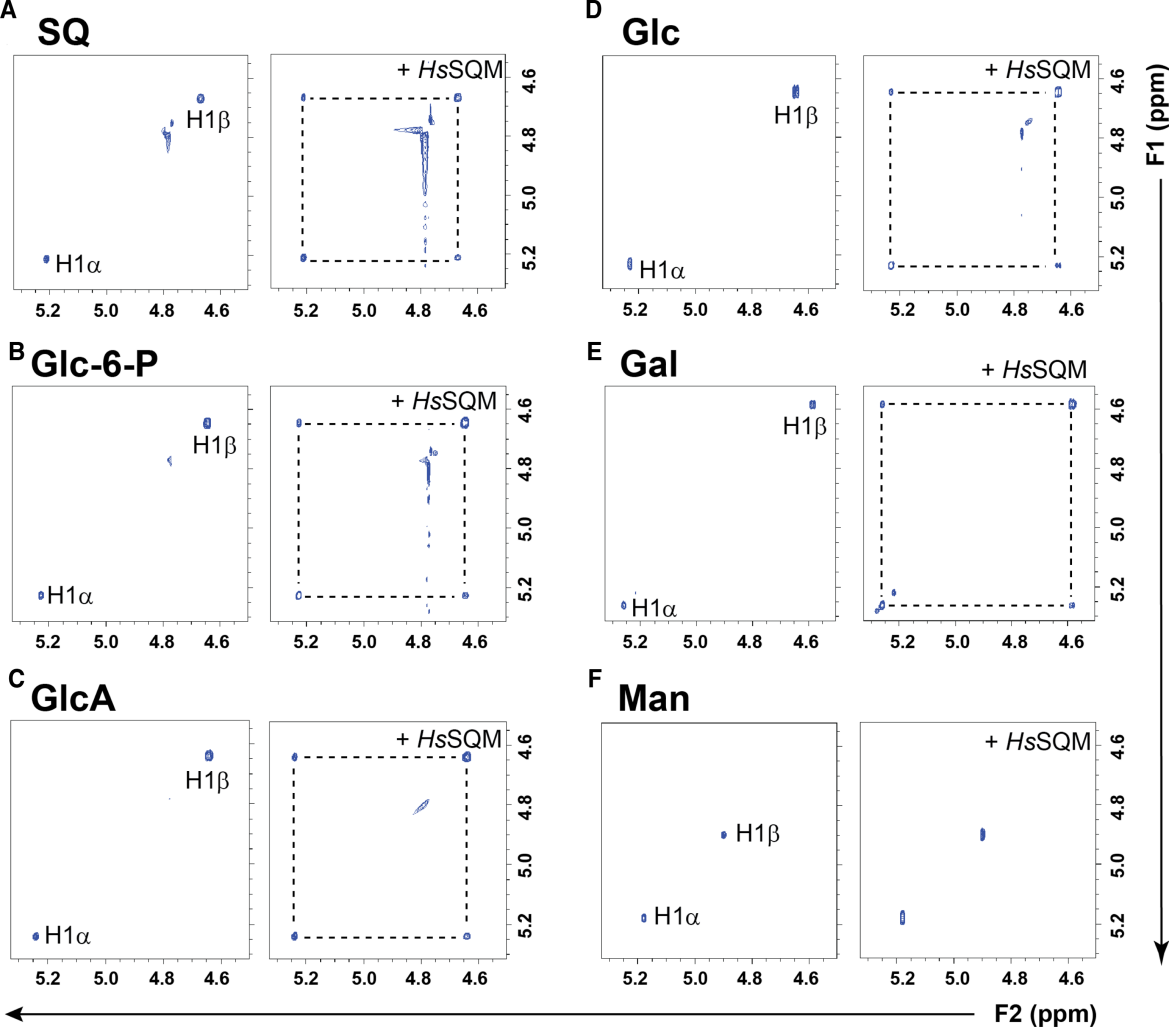
Excerpt showing anomeric regions of 2D ^1^H-^1^H EXSY plots of various hexoses alone and with *H. seropediacae* mutarotase. (**A**) Sulfoquinovose (SQ), (**B**) d-glucose-6-phosphate (Glc-6-P), (**C**) d-glucuronic acid (GlcA), (**D**) d-glucose (Glc), (**E**) d-galactose (Gal), and (**F**) d-mannose (Man). Hexoses are at 5 mM, 1.51 μM *Hs*SQM in 50 mM sodium phosphate, 150 mM NaCl (pD 7.5).

2D ^1^H-^1^H EXSY was used to assess whether *Hs*SQM can catalyze mutarotation. A 2D pulse sequence equivalent to that used for a 2D ^1^H-^1^H NOESY experiment was employed to provide a visual representation of the chemical exchange network [15,20]. A similar approach was utilized by Graille et al. [32] to study a hexose-6-phosphate mutarotase, building on work by Balaban and Ferretti [17] for the study of anomerization/isomerization of Glc-6-P by phosphoglucose isomerase using 2D ^31^P-^31^P EXSY, and earlier work by Kuchel et al. [16], who studied mutarotation catalyzed by porcine mutarotase using ^13^C-^13^C EXSY. In this approach, all nuclei are excited using a 90° excitation pulse, which is allowed to evolve over a *t*_1_ period. A second 90° pulse is then applied to rotate the *y*-component of magnetization onto the *z*-axis, and a mixing interval *τ*_mix_ allows magnetization transfer through chemical exchange. A final 90° pulse creates transverse magnetization in the *xy* plane, which is detected. Molecules that undergo chemical interconversion display cross-peak signals between a signal from the substrate along one axis of the 2D spectrum and the corresponding nucleus in the product on the other axis. Careful choice of mixing time to be shorter than that required for chemical exchange by spontaneous mutarotation can allow qualitative detection of enzyme-catalyzed mutarotation in a single NMR experiment. Figure 4A shows that a solution of SQ displays H1 of the α- and β-anomers as independent sets of signals, and upon the addition of *Hs*SQM, off-diagonal cross-peaks appear between the anomeric proteins. These data provide evidence that chemical exchange is occurring and that *Hs*SQM is catalyzing the mutarotation of SQ.

To qualitatively explore the substrate specificity of *Hs*SQM, we assessed the ability of the enzyme to catalyze mutarotation of other simple hexoses (Figure 4B–F). *Hs*SQM could catalyze the mutarotation of Glc-6-P and d-glucuronic acid, showing that the sulfonate group is not required and that *Hs*SQM can tolerate other anionic groups. A set of simple aldohexoses was also studied. *Hs*SQM catalyzed mutarotation of d-glucose and d-galactose, showing that the enzyme is tolerant of either stereochemistry at C4. However, using this pulse sequence, we could not detect *Hs*SQM-mediated mutarotation of d-mannose on the NMR time-scale, demonstrating the preference of *Hs*SQM for substrates with an equatorial C2-hydroxyl group.

**Figure 5.**
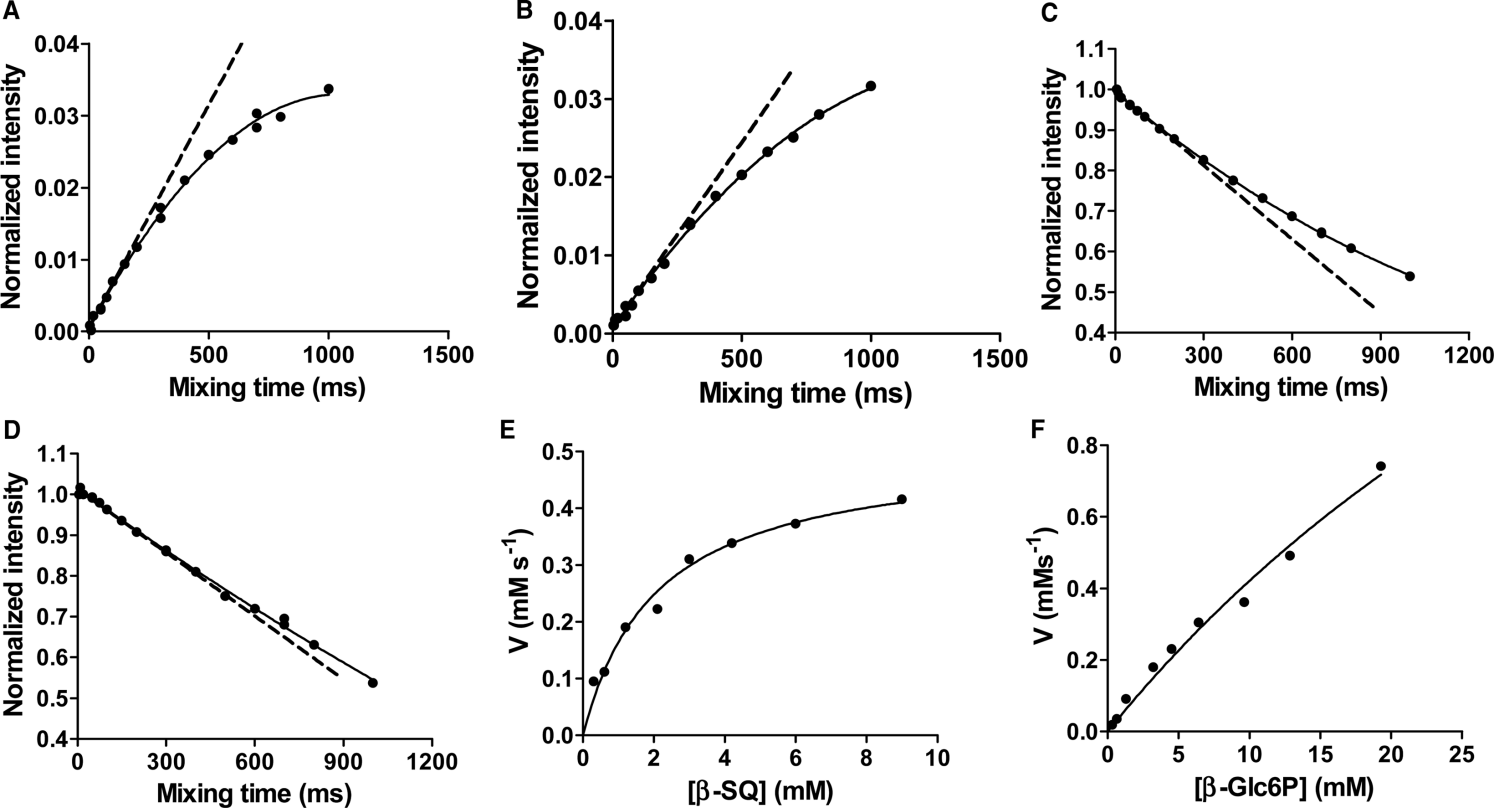
Kinetic analysis of *Hs*SQM by inversion recovery 1D ^1^H EXSY. Inversion recovery curves for 10 mM SQ or Glc-6-P corresponding to (**A**) α-SQ at 4.00 mM and (**B**) α-Glc-6-P at 3.57 mM. Inversion decay curves corresponding to (**C**) β-SQ at 6.00 mM and (**D**) β-Glc-6-P at 6.43 mM. Michaelis–Menten plots for (**E**) β-SQ and (**F**) β-Glc-6-P. Dashed lines indicate tangents to the fitted curve at *t* = 0.

While these experiments provide insights into the substrate specificity of *Hs*SQM, simple hexoses are not present at any appreciable concentration within bacterial cells, as shown for *E. coli* grown on various carbon sources [33]. Other than SQ, the main other physiologically plausible substrate for *Hs*SQM is Glc-6-P: an abundant primary metabolite produced by bacteria grown on glucose, glycerol, or acetate [33]. To explore the selectivity of *Hs*SQM for SQ and Glc-6-P, we used EXSY to determine kinetic parameters under conditions of equilibrium exchange. While it is possible to use 2D methods to determine rates, accurate determination requires *a priori* knowledge of optimum mixing times and a lengthy experiment, and so, we elected to use the alternative 1D EXSY approach. The measurement of the equilibrium-exchange rate constants was achieved by selective inversion of a distinctive signal from one anomer and then monitoring the return to equilibrium intensity of signals from both anomers. For the quantitative determination of rates, the selective irradiation must be of sufficient power and duration to invert the targeted magnetization, yet should not directly affect the equilibrium magnetization of the other spin population. Owing to the chemical exchange of the anomers, the inverted magnetization redistributes into the unirradiated site. The magnetization can then be sampled by normal methods. Figure 5A,B shows buildup curves of H5alpha or H1α arising from the irradiation of H5beta or H1β of SQ and Glc-6-P, respectively; and Figure 5C,D shows the respective decay curves from the same experiment for H1β. Also shown is the tangent at *τ*_mix_ = 0, which provides the exchange-rate constant. Figure 5E,F shows Michaelis–Menten plots for the conversion of the β-anomers of SQ and Glc-6-P, respectively. While SQ exhibited saturation kinetics, allowing calculation of *k*_cat_, *K*_M_, and *k*_cat_/*K*_M_ values, Glc-6-P did not and thus only *k*_cat_/*K*_M_ could be calculated. As the equilibrium concentrations of the two anomers of each substrate are known, these data allow calculation of kinetic parameters for the reverse reaction. The complete set of kinetic parameters for the forward and reverse mutarotation reactions are shown in Table 1 and reveals that *Hs*SQM displays an approximate 5-fold selectivity for SQ over Glc-6-P in terms of the *k*_cat_/*K*_M_ values.

**Table 1:** Michaelis–Menten kinetic parameters for *Hs*SQM-catalyzed mutarotation of SQ and Glc-6-P under conditions of equilibrium exchange

Substrate	*k*_cat_ (s^−1^)	*K*_M_ (mM)	*k*_cat_/*K*_M_ (M^−1^ s^−1^)
α-SQ	(3.08 ± 0.19) × 10^2^	1.00 ± 0.20	(3.07 ± 0.44) × 10^5^
β-SQ	(3.37 ± 0.20) × 10^2^	2.08 ± 0.34	(1.62 ± 0.18) × 10^5^
α-Glc-6-P	—	—	(5.87 ± 0.57) × 10^4^
β-Glc-6-P	—	—	(3.26 ± 0.31) × 10^4^

**Figure 6.**
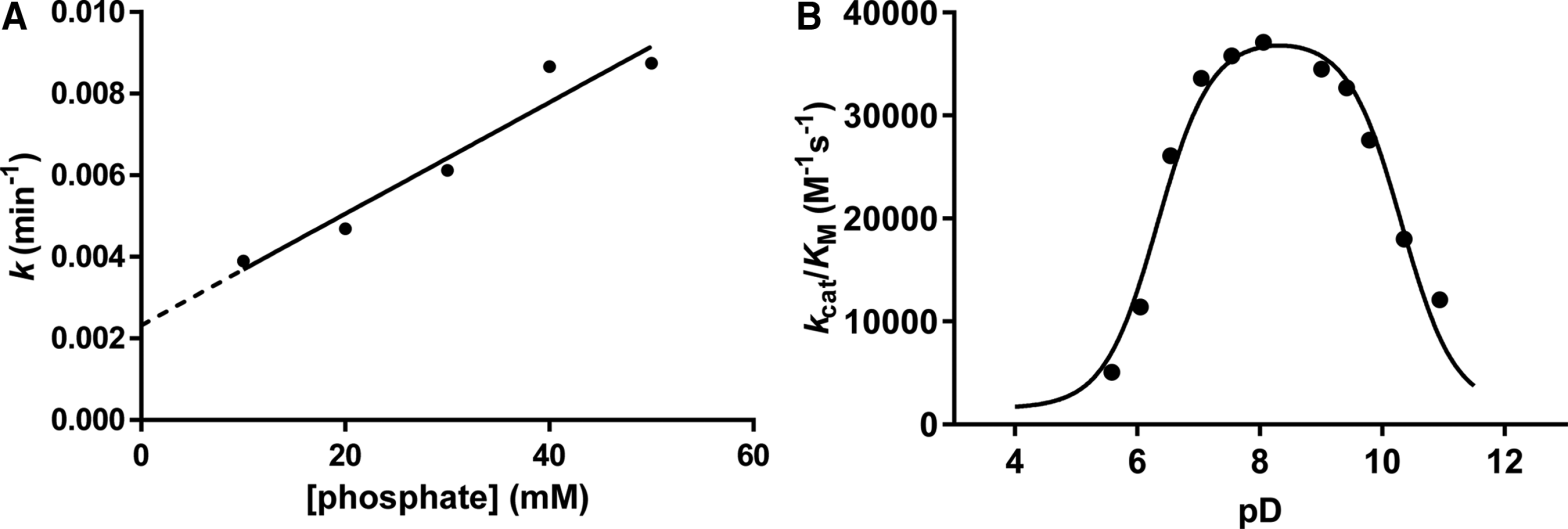
Spontaneous mutarotation of α-SQ and pD dependence of *Hs*SQM. (**A**) Plot of rates of spontaneous mutarotation of α-SQ versus phosphate buffer concentration at pseudo-constant ionic strength. Buffers consisted of 10–50 mM sodium phosphate and 2 M NaCl in D_2_O (pD 7.5). For comparison, *k* = 0.0073 min^−1^ in 50 mM sodium phosphate and 150 mM NaCl in D_2_O (pD 7.5). (**B**) pD dependence of *Hs*SQM activity for mutarotation of Glc-6-P. Data were fit to a bell-shaped curve leading to estimated p*K*_a_ values of 5.9 ± 0.1 and 9.9 ± 0.1.

To gain insights into the rate enhancement achieved by *Hs*SQM, we measured the rate of mutarotation of SQ by polarimetry using a coupled assay. Incubation of 4-nitrophenyl α-d-sulfoquinovoside with a retaining sulfoquinovosidase occurs with retention of configuration to afford α-SQ. Monitoring the rate of mutarotation of α-SQ in 50 mM phosphate buffer revealed a half-life of 50 min (Supplementary Figure S1). By comparison, mutarotation of α-Glc-6-P has been reported to occur at 25°C with a half-life of 6.0 s [34,35]. However, phosphate buffer is known to catalyze mutarotation [36]. To determine the rate of mutarotation in phosphate-free conditions, and to subsequently allow comparison with enzyme-catalyzed rates measured in D_2_O, we measured mutarotation rates of SQ in D_2_O at various concentrations of phosphate at pseudo-constant ionic strength achieved with 2 M NaCl (Figure 6A). Extrapolation of this data to [phosphate] = 0 mM gave a predicted mutarotation rate (*k*) of 3.87 × 10^−5 ^s^−1^ (0.00232 min^−1^) corresponding to a half-life of 299 min. Using the equilibrium constant *K*^eq^ = 1.5 allows the calculation of forward and reverse rates of *k*_1_ = 2.3 × 10^–5^ s^–1^ and *k*_–1_ = 1.5 × 10^–5^ s^–1^. A similar calculation for Glc-6-P mutarotation (*K*^eq^ = 1.8) using the published data gave forward and reverse rates of *k*_1_ = 7.7 × 10^–2^ s^–1^ and *k_−_*_1_ = 4.3 × 10^–2^ s^–1^; strictly speaking these ‘uncatalyzed’ rates are for $k_{\left(H_{2}O\right)}+k_{\left(H^{+}\right)}[H_{3}O^{+}]+k_{\left(HO^{-}\right)}[HO^{-}]$.

The above data allow us to calculate the catalytic proficiency of *Hs*SQM for catalyzing the mutarotation of SQ and Glc-6-P. The proficiency constants [*k*_uncat_/(*k*_cat_/*K*_M_)] for *Hs*SQM are 7.6 × 10^−11^ M for α-SQ and 1.3 × 10^−6^ M for α-Glc-6-P, revealing that *Hs*SQM binds the SQ transition state some 17 000-fold tighter than for Glc-6-P and is thus best considered a sulfoquinovose mutarotase. We note that the mutarotation rate for Glc-6-P was measured in H_2_O, whereas all other measurements were performed in D_2_O, thus a solvent isotope effect may somewhat confound this analysis; nonetheless, the broad conclusions still apply.

The pD dependence of activity for *Hs*SQM was determined using 1D ^1^H EXSY for Glc-6-P. At a concentration of 5 mM, Glc-6-P does not show saturation (unlike for SQ), and so rates measured at this concentration provide an estimate of *k*_cat_/*K*_M_. Figure 6 shows that the pD dependence of activity is bell-shaped, with a broad maximum of activity of pD 7–9. The simplest interpretation of these results is that it arises from ionization of two catalytically important residues with p*K*_a_ values of 6.3 ± 0.2 and 10.3 ± 0.2. The ionization of the acidic limb presumably reflections ionization of the general acid Glu254, and the basic limb ionization of the general base His92. By comparison, the intrinsic p*K*_a_ values for the ionizable side-chains of Glu and His are 4.5 and 6.4, respectively [37]. In both cases, and particularly the latter, these intrinsic p*K*_a_ values are perturbed, presumably because of the active-site environment including charge–charge and charge–dipole interactions, as well as desolvation effects [37]. The pD dependence of this enzyme is similar to the pH dependence of green pepper mutarotase [34], but broader than that of *E. coli* galactose mutarotase [38].

## Discussion

Many enzymes involved in the metabolism of free sugars are stereospecific for one anomer of their substrate. For example, yeast galactokinase specifically phosphorylates α-galactose to produce α-d-galactose-1-phosphate [39], and glucose dehydrogenase from *Bacillus megaterium* uses only β-d-glucose as a substrate [40]; yeast phosphoglucose isomerase only uses α-Glc-6-P as a substrate [41], and yeast phosphomannose isomerase is specific for α-d-mannose-6-phosphate [42]. For these enzymes, access to the appropriate substrate anomer would be rate-limiting *in vivo* if the process relied on spontaneous mutarotation. Mutarotases ensure that there is rapid equilibration between anomers to eliminate this metabolic bottleneck. While some mutarotases have been linked to specific metabolic pathways [6], it has been difficult to assign many others to a single substrate or metabolic process [32]. It is unclear if the inherent promiscuity of many mutarotases confers an advantage to their hosts or simply imposes no cost to fitness.

Sulfo-EMP and sulfo-ED gene clusters both encode putative mutarotases, homologous to *Hs*SQM, that are up-regulated in response to growth on SQ [1,2], suggesting that their primary function is as an SQ mutarotase. We show here that *Hs*SQM from the sulfo-ED cluster of *H. seropedicaea* can catalyze SQ mutarotation with a greater *k*_cat_/*K*_M_ value than for the possible alternative substrate Glc-6-P, which is itself an important metabolite that feeds into the EMP and ED glycolytic pathways, as well as the pentose phosphate pathway. It is possible that the activity of the enzyme on Glc-6-P is advantageous to a bacterium transitioning between growth on SQ and growth on Glc. Furthermore, due to the broad substrate tolerance of many mutarotases, it is possible that other enzymes may play a role in catalyzing SQ mutarotation within the cell, though any enzyme that does so is unlikely to be transcriptionally regulated by SQ concentration as members of sulfoglycolytic operons are.

The substrate specificity of *Hs*SQM bears similarities to the aldose-1-epimerase from *E. coli* (galM), which is tolerant to functional group changes at C6, and stereochemistry changes at C4 (Gal) but not at C2 (Man) [9]. On the other hand, it is distinguished from yeast ymr099c, which encodes a hexose-6-phosphate mutarotase with activity on Glc-6-P, Gal-6-P, and Man-6-P and thus tolerates stereochemical inversion at C2 [32]. Sequence alignments and structural modeling reveal that *Hs*SQM likely acts through a mechanism conserved with all other mutarotases. The substrate tolerance of *Hs*SQM for substituent variation at C6 lies in contrast with *E. coli* SQase, which exhibited negligible activity on α-glucosides due to its specialized set of conserved residues that recognize the sulfonate group of SQ glycosides [4].

The effectiveness of an enzyme as a catalyst can be quantified by measuring the rate enhancement it provides relative to the rate of the uncatalyzed reaction [43]. This work reveals that uncatalyzed SQ mutarotation is a relatively slow reaction, with the rate for α-SQ some 3400-fold lower than that of α-Glc-6-P. On the other hand, *Hs*SQM catalyzes mutarotation with a *k*_cat_/*K*_M_ value ∼5-fold higher than for Glc-6-P. Combining these values reveals that *Hs*SQM is ∼17 000-fold more proficient as a catalyst for the mutarotation of SQ and Glc-6-P. As enzymes achieve their rate enhancement through selective stabilization of the transition state relative to the ground state, these data suggest that the affinity for the transition state of SQ mutarotation is ∼17 000-fold greater than that for Glc-6-P mutarotation. As has been noted by others, the unusually high rate of mutarotation of Glc-6-P is much greater than that of other hexoses [44] and appears to be a result of neighboring group participation by the pendant phosphate group [36]; our data suggest that the sulfonate group of SQ does not provide neighboring group participation.

Understanding the precise role of SQMs in the sulfo-EMP and sulfo-ED pathways will require knowledge of the substrate specificities for upstream and downstream processes. Upstream processes include SQ importers and SQases. For organisms grown on a mixture of SQ anomers, the SQ importers may exhibit a preference for only one SQ anomer; an SQM may be required for re-establishing an equilibrium mixture of SQ anomers. A pertinent example is that of red blood cells in which it is known that glucose importers exhibit a preference for α-glucose [45], and a potential role for glucomutarotase in a permease system involved in re-establishing this equilibrium has been advanced [34]. For cells grown on SQ glycosides, SQ is released by action of SQases; the immediate product is α-SQ, and SQMs may be required to enhance the conversion of α-SQ to β-SQ, to match the preference of a downstream enzyme, or to act in the reverse direction to ‘rescue’ β-SQ that would otherwise accumulate as a result of the spontaneous mutarotation of α-SQ. Downstream enzymes include SQ isomerases and SQ dehydrogenases. As yet the anomeric preference, if any, of these enzymes is unknown. The NMR EXSY experiments used here to probe SQM activity, which build on pioneering work reaching back many decades, could be of use for studying the substrate preferences of the downstream enzymes, and in so doing could help provide the biological context for the SQMs.

## Abbreviations

DHAP: dihydroxyacetone phosphate
DHPS: 2,3-dihydroxypropanesulfonate
DSS: (4,4-dimethyl-4-silapentane-1-sulfonic acid)
ED: Entner–Doudoroff
EMP: Embden–Meyerof–Parnas
EXSY: exchange spectroscopy
Gal: galactose
Glc: glucose
GlcA: glucuronic acid
*Hs*SQM: *Herbaspirillum seropedicaea* sulfoquinovose mutarotase
Man: mannose
NMR: nuclear magnetic resonance
PNPSQ: 4-nitrophenyl α-d-sulfoquinovoside
SL: sulfolactate
SLA: sulfolactaldehyde
SQ: sulfoquinovose
SQase: sulfoquinovosidase
SQGro: α-sulfoquinovosyl glycerol
SQM: sulfoquinovose mutarotase
