# Supplementary material for: Discovery and characterization of a sulfoquinovose mutarotase using kinetic analysis at equilibrium by exchange spectroscopy
Source: Biochem J. 2018 Apr 16;475(7):1371–83. doi: 10.1042/BCJ20170947 (PMC5902678; doi:10.1042/BCJ20170947)

SUPPORTING INFORMATION

Discovery and characterization of a sulfoquinovose mutarotase using kinetic analysis at equilibrium by exchange spectroscopy

Palika Abayakoon, James P. Lingford, Yi Jin, Christopher Bengt, Gideon J. Davies, Shenggen Yao,* Ethan D. Goddard-Borger,* Spencer J. Williams*

Contents

*Hs*SQM plasmid and sequence data 2
Table S1. Pairwise sequence comparisons of *Hs*SQM with various hexose mutarotases.* 3
Figure S1. Mutarotation of SQ monitored by polarimetry. 4
1D ^{1}H NMR spectra 5
  Sulfoquinovose (600 MHz, 15 mM, D_2O) 5
  Glucose-6-phosphate (600 MHz, 15 mM, D_2O) 5
2D ^{1}H-^{1}H NOESY spectra with 1D spectrum projected onto F1 and F2 axes 6
  Sulfoquinovose (5 mM), no enzyme 6
  Sulfoquinovose (5 mM), with HsSQM 6
  D-Glucose-6-phosphate (5 mM), no enzyme 7
  D-Glucose-6-phosphate (5 mM), with HsSQM 7
  D-Glucuronic acid (5 mM), no enzyme 8
  D-Glucuronic acid (5 mM), with HsSQM 8
  D-Glucose (5 mM), no enzyme 9
  D-Glucose (5 mM), with HsSQM 9
  D-Galactose (5 mM), no enzyme 10
  D-Galactose (5 mM), with HsSQM 10
  D-Mannose (5 mM), no enzyme 11
  D-Mannose (5 mM), with HsSQM 11

*Hs*SQM plasmid and sequence data

plasmid	resistance	details
pET29-HsSQM	Kan	C-terminal His_6-tagged *Herbaspirillum seropedicae* strain AU14040 sulfoquinovose mutarotase (WP_069374721.1)

pET29-HsSQM

CDS:
ATGTCGGCAGCTCTTGCGTCCCTGACACTTGCCCAAGGACCGTGGCAAGTGCGCGTGCTGCCTGCACTGGGCGGTGCGATTGC
AAGCGCCAGCTGGCGTGGTCAGCCGGTGCTGCGCCCGAGCGTCGAGGCACAGCTGCAGCAGGGCCTGGTTAGACGCTCTGCGT
GCTATCCGCTGCTGCCGTTTAGTAACCGTATCGGAAACGCGCAATTCGCCTTTGATGGCCAGACATACGCGCTGATTGCCAAC
TTTGACAACGAACCGCACGCTATCCACGGGCTGGGCTTTCAGCGCGCGTGGCAGGTTCAGTCTAGCTCGGCGGAAAGTCTTAC
TATGCAGCTCACCCACGCCTCGCCGAGCCCGGGTCAGTGGCCGTTTGCCTTGCGTGCCACCCAGGTGATTGCCATCGAAGGCG
ACGATCTGGTGCTGCGCCTGGAAGTGGAAAATACCGATCATCGCCGCGCCCCATGCGGCCTGGGCTGGCATCCGTTTTTCCG
CTGGATAGCGCGGCGCAGCCGACCCAGCTGCAGACCCATTGGCAAGCGATGCTGGTAAACGGGCCAGACAAACTTCCGTGCGG
CAGCACGGCCCCTCCGGATACGACCCAACTGGATACCTTAGTGATTGATAATTGCTTTACCGGCTGGAGCGGCCAGGCCGTCG
TTACCGGCCCGCATCATCGTATTACCCTGACAGCGAGCCCGACCCTGCGTTGCGCGGTGCTGTTTCGCCCGCCGGGGCAGCCG
TTCTTCGCGTTTGAACCGGTGAGCCATCCGAACAATGCCCTGCACGGCGTTGCGCCGGCGATGCACATCCTGGAACCCGGTCA
GTGCCTGGCGGGCGAAATGCGCCTGAGCCTGTCGACCGCTCCGAGCATTCTGGCGGCCGCACTCGAGCACCACCACCACCACC
ACTGA

Translation:
MSAALASLTLAQGPWQVRVLPALGGAIASASWRGQPVLRPSVEAQLQQGLVRRSACYPLL
PFSNRIGNAQFAFDGQTYALIANFDNEPHAIHGLGFQRAWQVQSSSAESLTMQLTHASPS
PGQWPFALRATQVIAIEGDDLVLRLEVENTDHRRAPCGLGWHPFFPLDSAAQPTQLQTHW
QAMLVNGPDKLPCGSTAPPDTTQLDTLVIDNCFTGWSGQAVVTGPHHRITLTASPTLRCA
VLFRPPGQPFFAFEPVSHPNNALHGVAPAMHILEPGQCLAGEMRLSLSTAPSILAAALEH
HHHHH*

Table S1. Pairwise sequence comparisons of HsSQM with various hexose mutarotases.[*]

H. seropediaceae	*P. putida*	*E. coli*	*L. lactis*	*S. cerevisiae*	
***Hs*SQM**<br>WP_069374721.1	**PpSQ_00415**<br>KHL76357.1	**YihQ**<br>NP_418315.3	**GalM**<br>WP_039116219.1	**YMR099C**<br>DAA09996.1	**% identity (%similarity)**
	35<br>(49)	19<br>(38)	13<br>(26)	13<br>(28)	**HsSQM**
		19<br>(37)	12<br>(24)	15<br>(29)	**PpSQ_00415**
			12<br>(29)	11<br>(23)	**YihQ**
				14<br>(23)	**GalM**
					**YMR099C**

[*] Calculated using the "Sequence Manipulation Suite" and the following groupings of similar amino acids: GAVLI, FYW, CM, ST, KRH, DENQ, P.

See: http://www.bioinformatics.org/sms2/ident_sim.html, Stothard, P. *Biotechniques*, 2000, 28, 1102–1104.

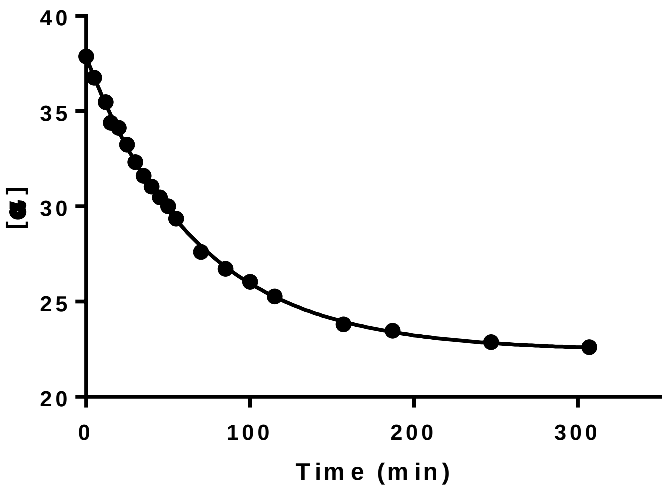

Figure S1. Mutarotation of SQ monitored by polarimetry.

4-Nitrophenyl α-D-sulfoquinovoside in 50 mM sodium phosphate, 150 mM NaCl in H_2O (pH 7.1) was treated with sulfoquinovosidase, and after 5 min, the specific rotation was monitored as a function of time. pH 7.1, 26±1 °C.

1D ^{1}H NMR spectra

Sulfoquinovose (600 MHz, 15 mM, D_2O)

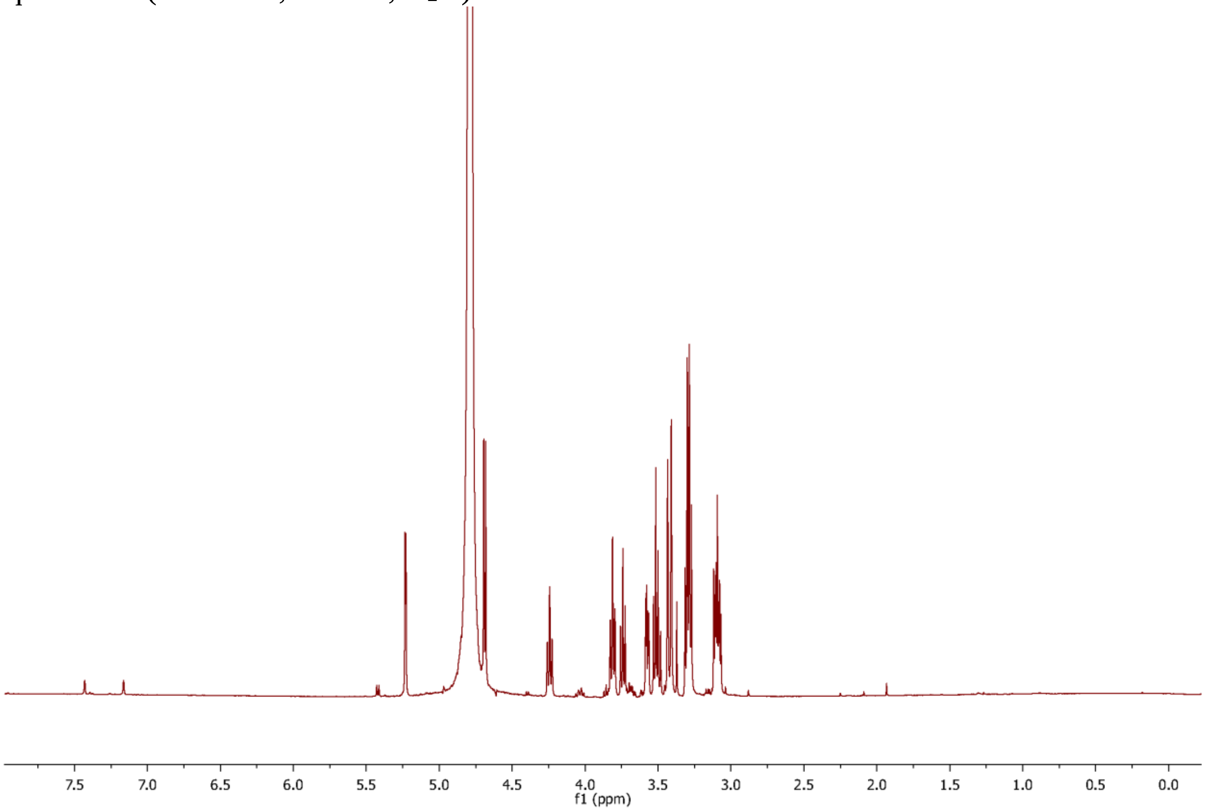

Glucose-6-phosphate (600 MHz, 15 mM, D_2O)

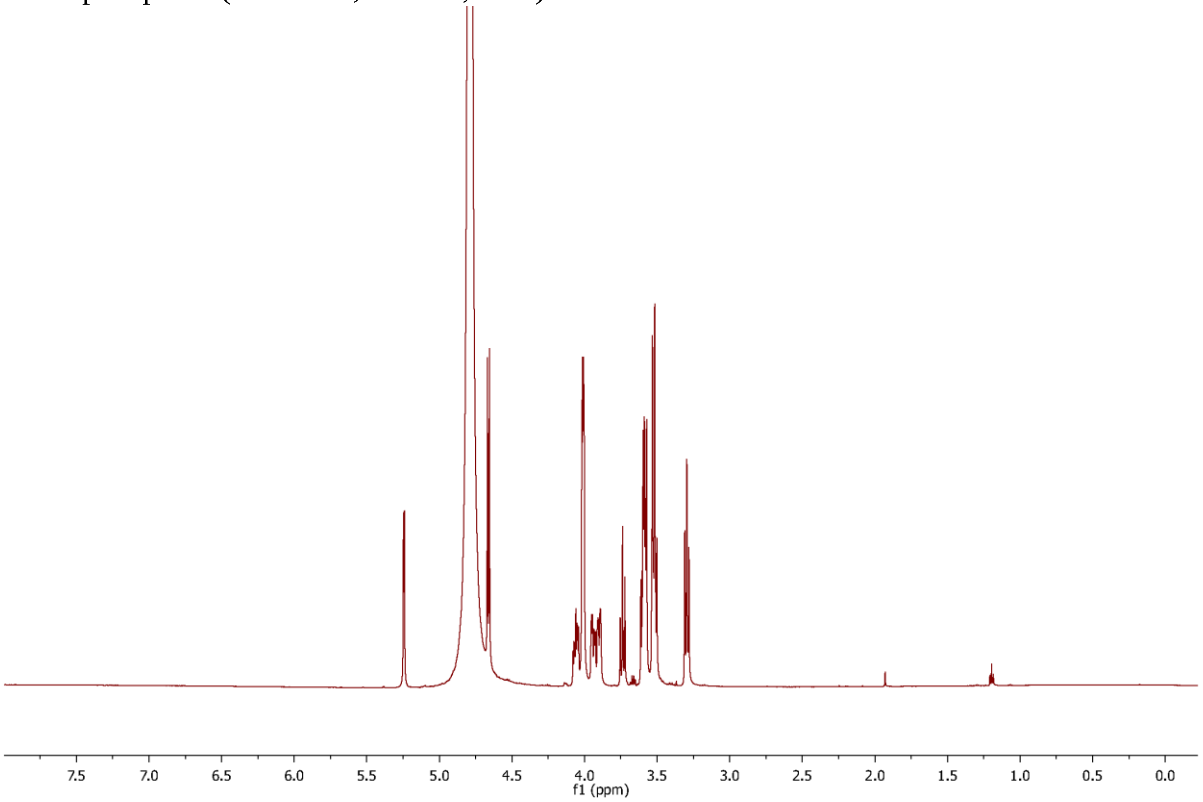

2D ^{1}H-^{1}H NOESY spectra with 1D spectrum projected onto F1 and F2 axes

Sulfoquinovose (5 mM), no enzyme

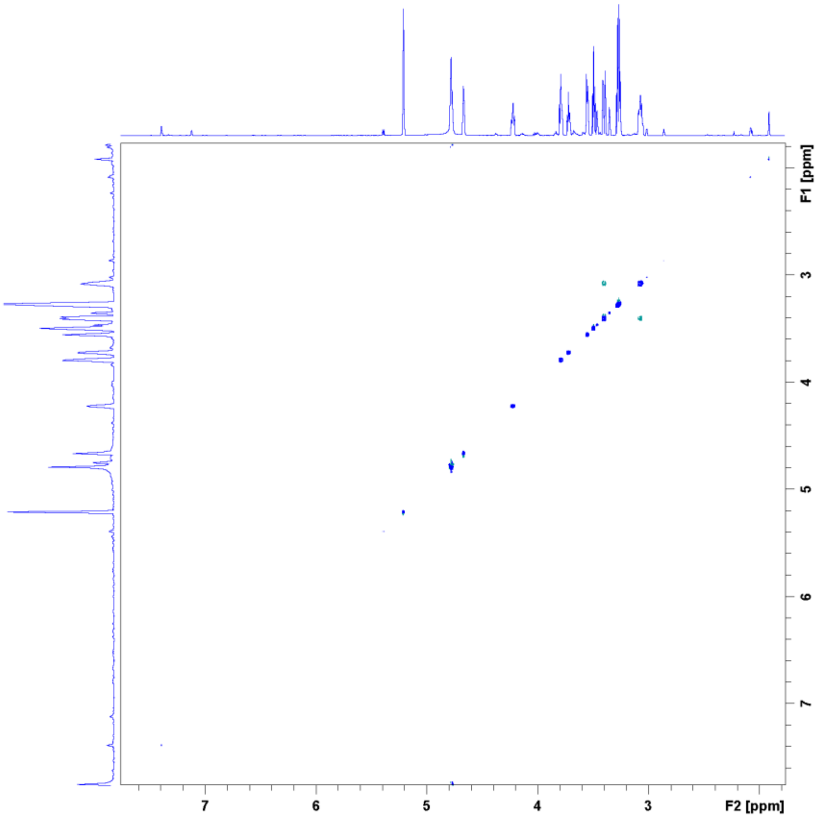

Sulfoquinovose (5 mM), with *Hs*SQM

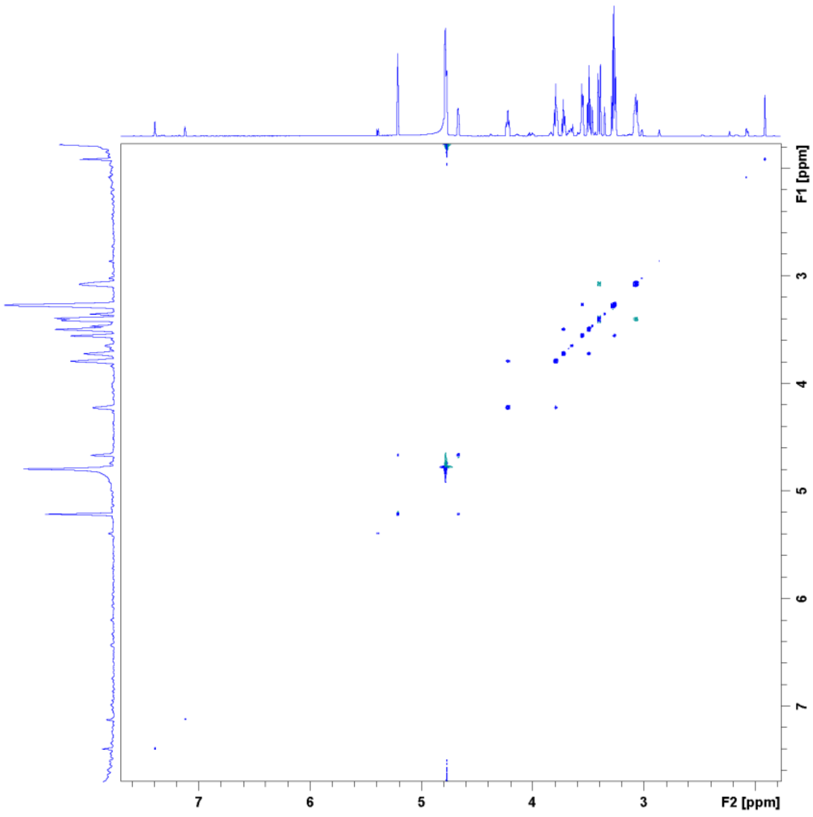

D-Glucose-6-phosphate (5 mM), no enzyme

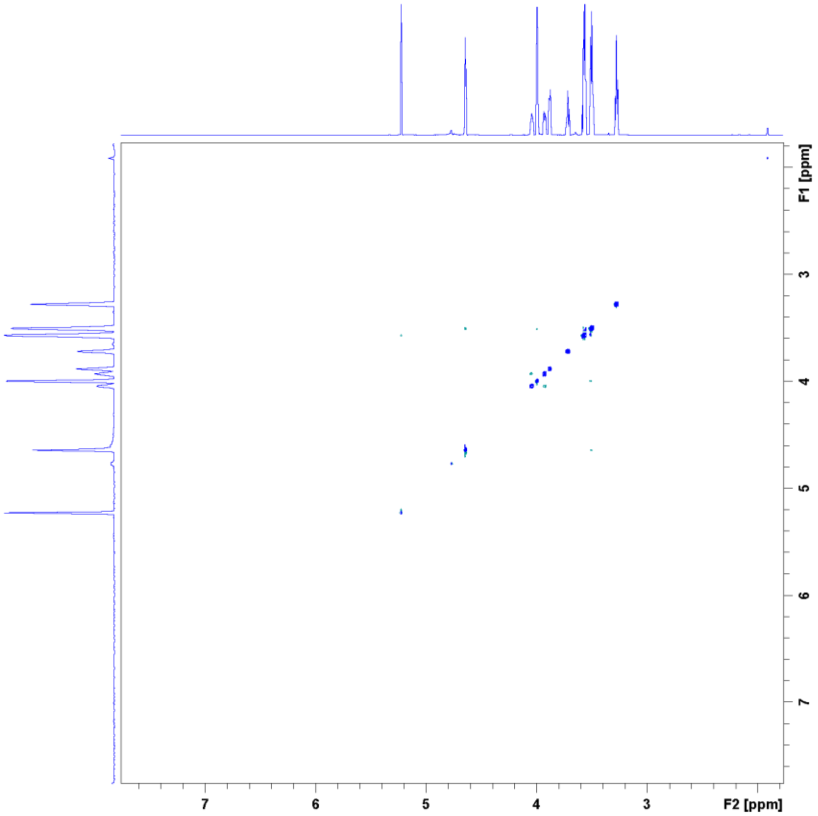

D-Glucose-6-phosphate (5 mM), with *Hs*SQM

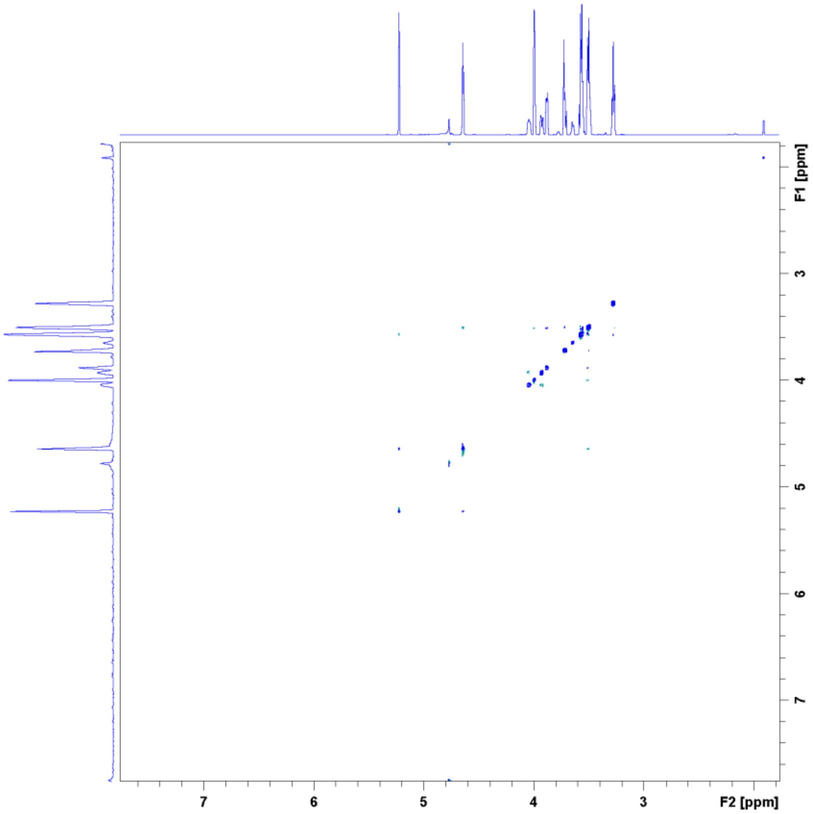

D-Glucuronic acid (5 mM), no enzyme

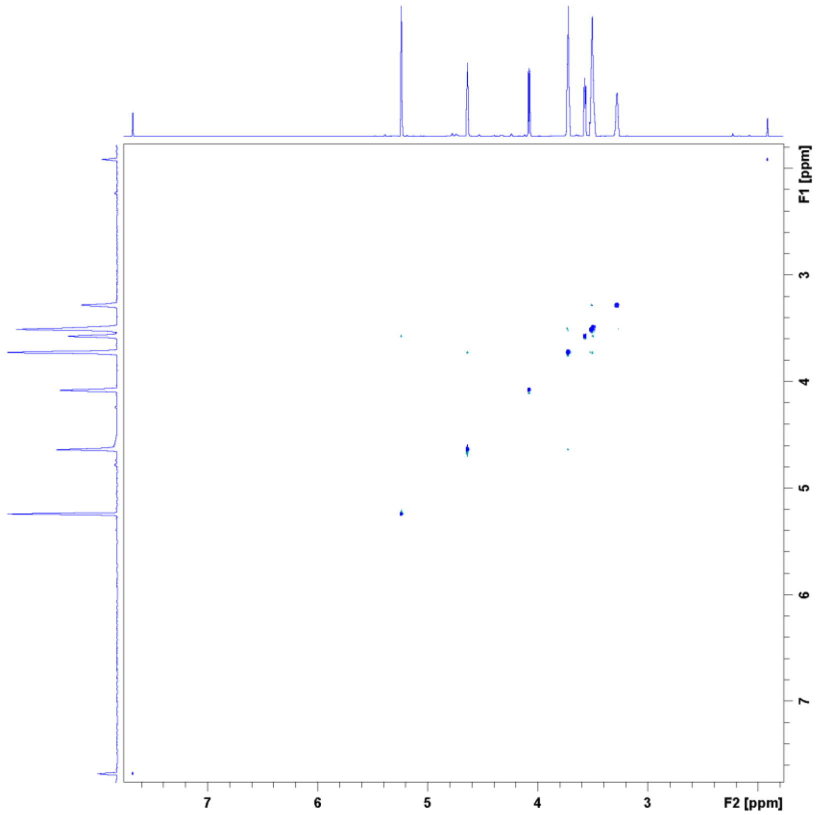

D-Glucuronic acid (5 mM), with *Hs*SQM

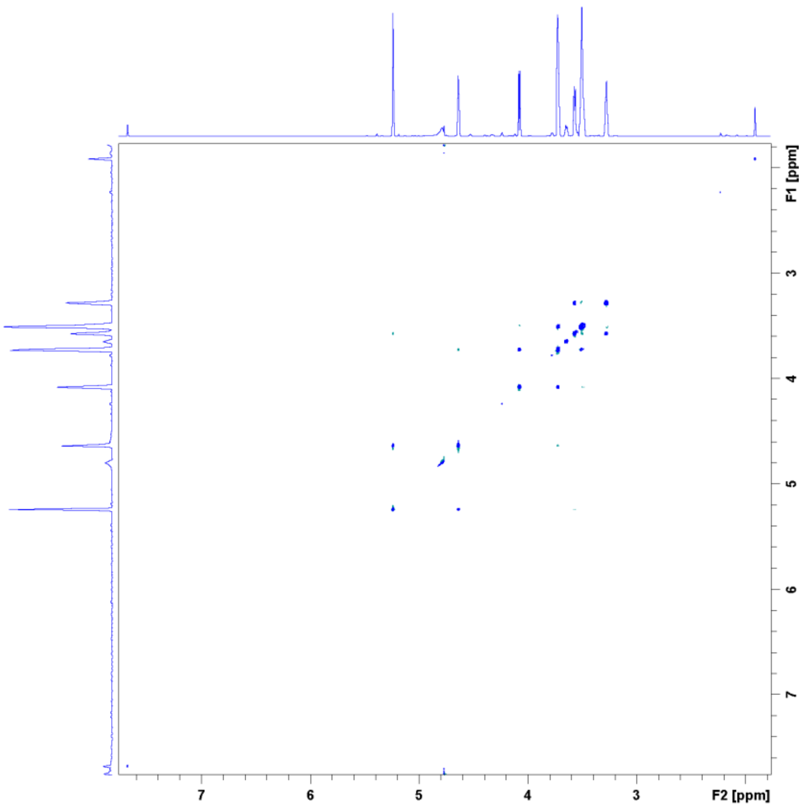

D-Glucose (5 mM), no enzyme

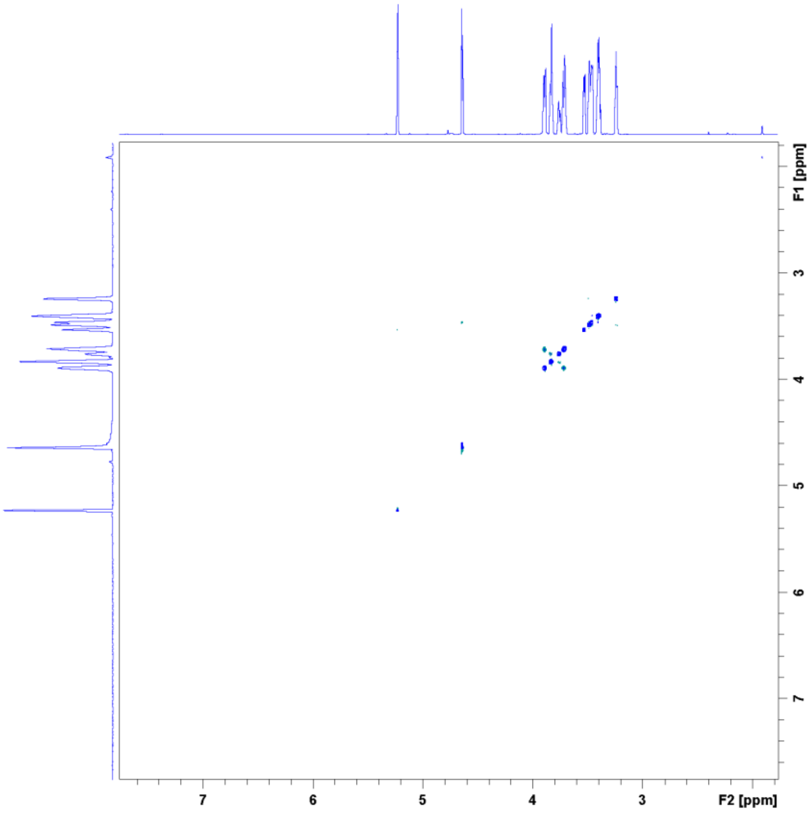

D-Glucose (5 mM), with *Hs*SQM

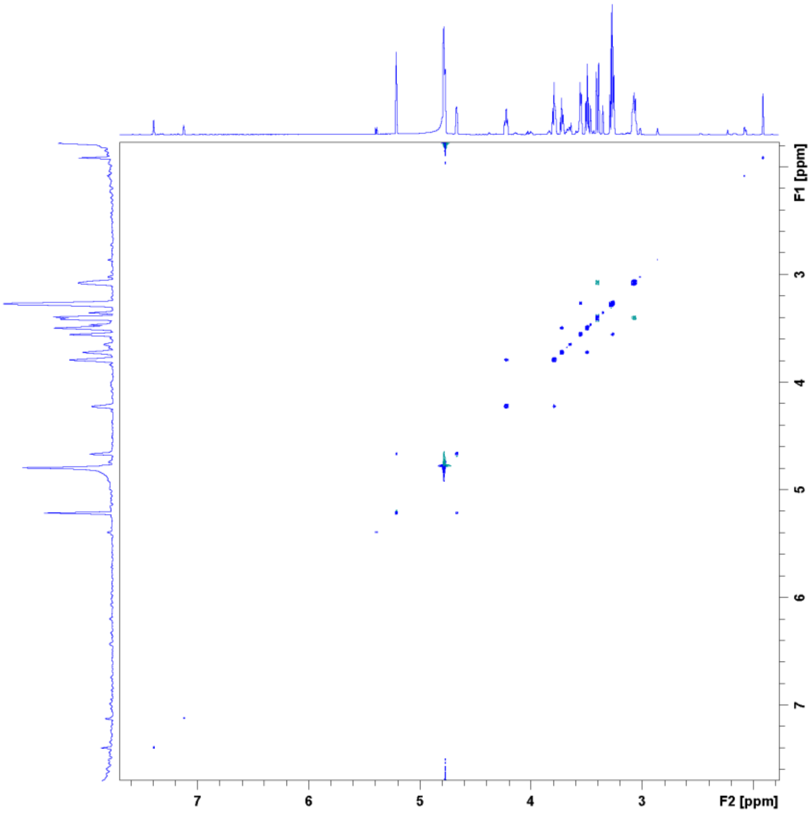

D-Galactose (5 mM), no enzyme

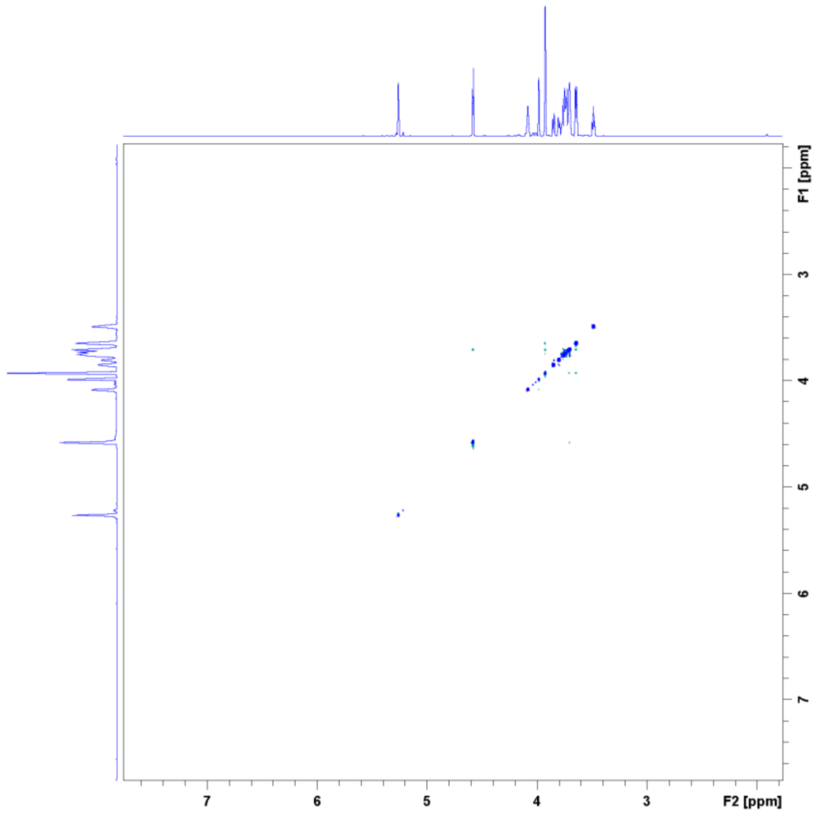

D-Galactose (5 mM), with *Hs*SQM

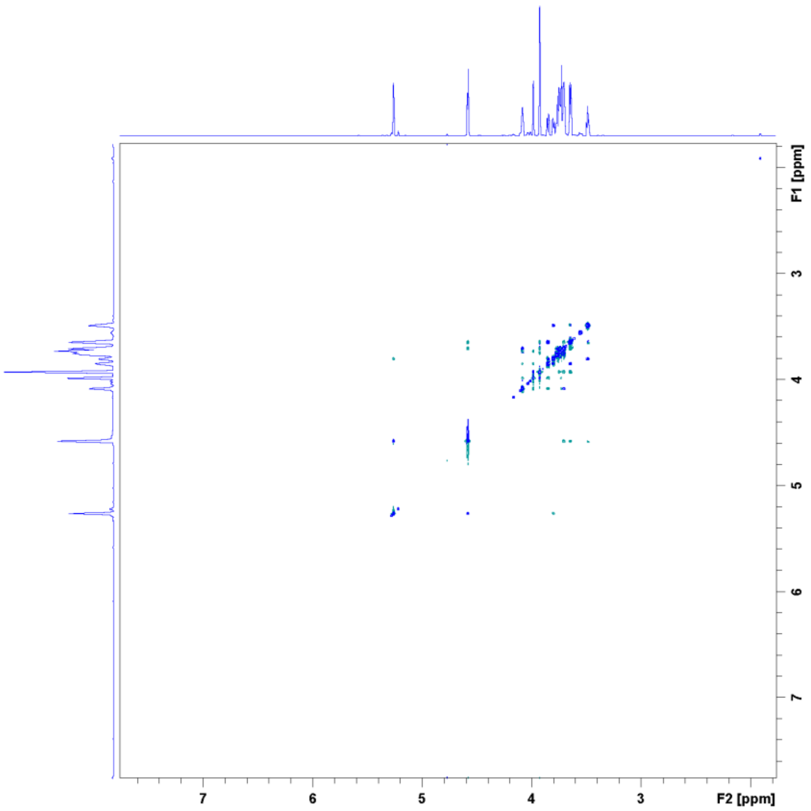

D-Mannose (5 mM), no enzyme

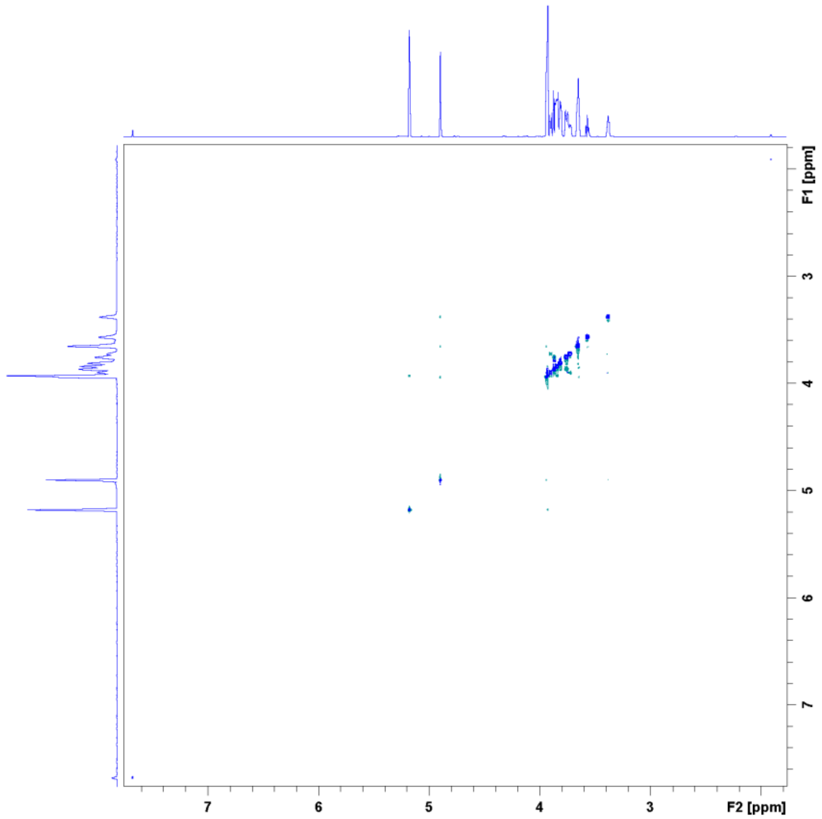

D-Mannose (5 mM), with *Hs*SQM

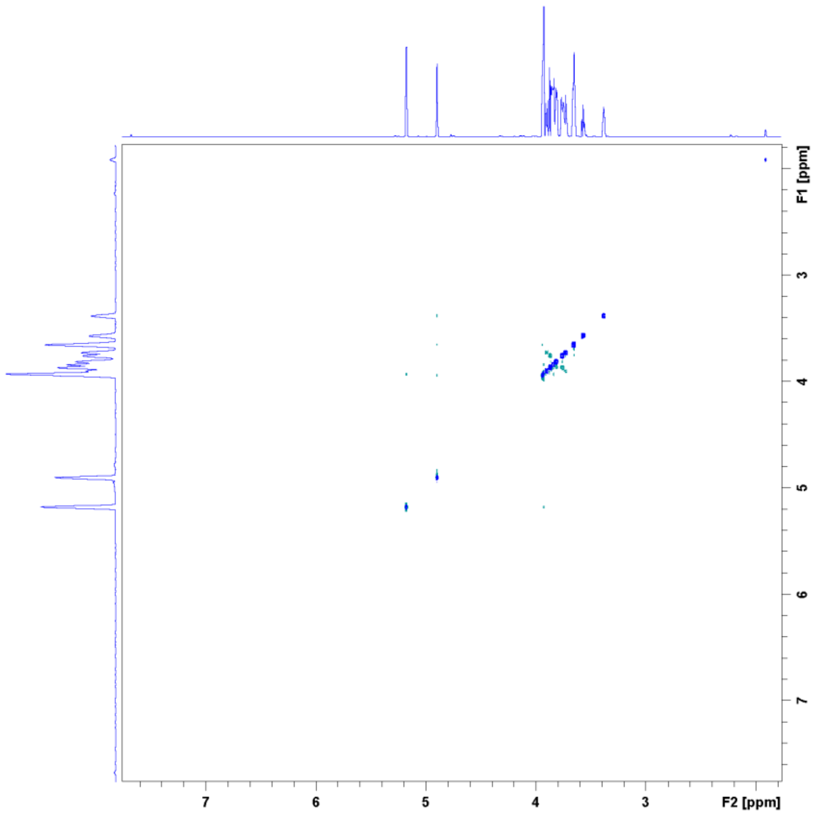

Supplement: Supplementary Figures and Table [file BCJ-475-1371-s1.pdf]
